# Positive Periodic Solutions of an Epidemic Model with Seasonality

**DOI:** 10.1155/2013/470646

**Published:** 2013-11-10

**Authors:** Gui-Quan Sun, Zhenguo Bai, Zi-Ke Zhang, Tao Zhou, Zhen Jin

**Affiliations:** ^1^Complex Sciences Center, Shanxi University, Taiyuan 030006, China; ^2^School of Mathematical Sciences, Shanxi University, Taiyuan, Shanxi 030006, China; ^3^Department of Mathematics, North University of China, Taiyuan, Shanxi 030051, China; ^4^Department of Applied Mathematics, Xidian University, Xi'an, Shaanxi 710071, China; ^5^Institute of Information Economy, Hangzhou Normal University, Hangzhou 310036, China; ^6^Web Sciences Center, University of Electronic Science and Technology of China, Chengdu, Sichuan 610054, China; ^7^Department of Physics, University of Fribourg, Chemin du Musée 3, 1700 Fribourg, Switzerland

## Abstract

An SEI autonomous model with logistic growth rate and its corresponding nonautonomous model are investigated. For the autonomous case, we give the attractive regions of equilibria and perform some numerical simulations. Basic demographic reproduction number *R*_*d*_ is obtained. Moreover, only the basic reproduction number *R*_0_ cannot ensure the existence of the positive equilibrium, which needs additional condition *R*_*d*_ > *R*_1_. For the nonautonomous case, by introducing the basic reproduction number defined by the spectral radius, we study the uniform persistence and extinction of the disease. The results show that for the periodic system the basic reproduction number is more accurate than the average reproduction number.

## 1. Introduction

Bernoulli was the first person to use mathematical method to evaluate the effectiveness of inoculation for smallpox [1–6]. Then in 1906, Hawer studied the regular occurrence of measles by a discrete-time model. Moreover, Ross [3, 4] adopted the continuous model to study the dynamics of malaria between mosquitoes and humans in 1916 and 1917. In 1927, Kermack and McKendrick [5, 6] extended the above works and established the threshold theory. So far, mathematical models have gotten great development and have been used to study population dynamics, ecology, and epidemic, which can be classified in terms of different aspects. From the aspect of the incidence of infectious diseases, there are bilinear incidence, standard incidence, saturating incidence, and so on. According to the type of demographic import, the constant import, the exponential import, and the logistic growth import are the most common forms. The simple exponential growth models can provide an adequate approximation to population growth for the initial period. If no predation or intraspecific competition for populations is included, the population can continue to increase. However, it is impossible to grow immoderately due to the intraspecific competition for environmental resources such as food and habitat. So, for this case, logistic model is more reasonable and realistic which has been adopted and studied [7–18]. Moreover, due to its rich dynamics, the logistic models have been applied to many fields. Fujikawa et al. [9] applied the logistic model to show *Escherichia coli* growth. Invernizzi and Terpin [14] used a generalized logistic model to describe photosynthetic growth and predict biomass production. Min et al. [15] used logistic dynamics model to describe coalmining cities' economic growth mechanism and sustainable development. There is a good fit in simulating the coalmining cities' growth and development track based on resource development cycle. Banaszak et al. [17] investigated logistic models in flexible manufacturing, and Brianzoni et al. [18] studied a business-cycle model with logistic population growth. Muroya [13] investigated discrete models of non-autonomous logistic equations. As a result, this paper builds an SEI ordinary differential model with the logistic growth rate and the standard incidence.

For general epidemic models, we mainly study their threshold dynamics, that is, the basic reproduction number which determines whether the disease can invade the susceptible population successfully. However, for the system with logistic growth rate, besides the basic reproduction number, the qualitative dynamics are controlled by a demographic threshold *R*_*d*_ which has a similar meaning and is called as the basic demographic reproduction number. If *R*_*d*_ > 1, the population grows; that is, a critical mass of individuals for the disease to spread may be supported. If *R*_*d*_ < 1, the population will not survive; that is, not enough mass of individuals may be supported for the disease to spread. For this case, the dynamical behavior of disease will be decided by two thresholds *R*_0_ and *R*_*d*_.

It is well known that many diseases exhibit seasonal fluctuations, such as whooping cough, measles, influenza, polio, chickenpox, mumps, and rabies [19–22]. Seasonally effective contact rate [22–26], periodic changing in the birth rate [27], and vaccination program [28] are often regarded as sources of periodicity. Seasonally effective contact rate is related to the behavior of people and animals, the temperature, and the economy. Due to the existence of different seasons, people have different activities which may lead to a different contact rate. Because of various factors, the economy in a different season has a very big difference. Therefore, this paper studies the corresponding non-autonomous system which is obtained by changing the constant transmission rate into the periodic transmission rate. Seasonal transmission is often assumed to be sinusoidal (cosine function has the same meaning), such that *λ*(*t*) = *λ*(1 + *η*sin(*πt*/*b*)) where *η* is the amplitude of seasonal variation in transmission (typically referred to as the “strength of seasonal forcing”) and 2*b* is the period, which is a crude assumption for many infectious diseases [29–31]. When *η* = 0, there is no nonseasonal infections. Motivated by biological realism, some recent papers take the contact rate as *λ*(*t*) = *λ*(1 + *η* term(*t*)), where term is a periodic function which is +1 during a period of time and −1 during other time. More natural term can be written as *λ*(*t*) = *λ*(1 + *η*)^term(*t*)^ [29]. Here, we take the form *β*(*t*) = *a*[1 + *b*sin(*πt*/10)].

The paper is organized as follows. In Section 2, we introduce an autonomous model and analyze the equilibria and their respective attractive region. In Section 3, we study the non-autonomous system in terms of global asymptotic stability of the disease-free equilibrium and the existence of positive periodic solutions. Moreover, numerical simulations are also performed. In Section 4, we give a brief discussion.

## 2. Autonomous Model and Analysis

### 2.1. Model Formulation

The model is a system of SEI ordinary differential equations, where *S* is the susceptible, *E* is the exposed, *I* is the infected, and *N* = *S* + *E* + *I*. This system considers the logistic growth rate and the standard incidence which is fit for the long-term growth of many large populations. The incubation period is considered for many diseases which do not develop symptoms immediately and need a period of time to accumulate a pathogen quantity for clinical outbreak, such as rabies, hand-foot-mouth disease, tuberculosis, and AIDS [22, 32]. The model we employ is as follows:

(1) $$\begin{array}{c} \frac{dS}{dt}=rN\left(1-\frac{N}{k}\right)-\frac{\beta SI}{N}-mS,\\ \frac{dE}{dt}=\frac{\beta SI}{N}-\sigma E-mE,\\ \frac{dI}{dt}=\sigma E-mI-\mu I, \end{array}$$

where all parameters are positive whose interpretations can be seen in Table 1.

Noticing the equations in model (1), we have

(2) $$\begin{array}{c} \frac{dN}{dt}=rN\left(1-\frac{N}{k}\right)-mN-\mu I. \end{array}$$

When there exists no disease, we have

(3) $$\begin{array}{c} \frac{dN}{dt}=rN\left(1-\frac{N}{k}\right)-mN=\left[r\left(1-\frac{N}{k}\right)-m\right]N. \end{array}$$

Let *R*_*d*_ = *r*/*m*, if *R*_*d*_ > 1, *N* → *N* ^0^ = (1 − *m*/*r*)*k* for *N*(0) > 0, as *t* → +*∞*; that is, the population will grow and tend to a steady state *N* ^0^. If *R*_*d*_ < 1, then *dN*/*dt* < 0 which will cause the population to disappear. Thus, *R*_*d*_ is the basic demographic reproduction number. From the above equation, the feasible region can be obtained: *X* = {(*S*, *E*, *I*) | *S*, *E*, *I* ≥ 0, 0 ≤ *S* + *E* + *I* ≤ (1 − *m*/*r*)*k*}, where *r* > *m*.

**Theorem 1**

The region *X* is positively invariant with respect to system (1).

### 2.2. Dynamical Analysis

Let the right hand of system (1) to be zero; it is easy to see that system (1) has three equilibria:

(4) $$\begin{array}{c} O=\left(0,0,0\right),\\ E_{0}=\left(\left(1-\frac{m}{r}\right)k,0,0\right),\\ E_{*}=\left(S^{*},E^{*},I^{*}\right), \end{array}$$

where *O* is the origin, *E*_0_ is the disease-free equilibrium, and *E*_∗_ is the endemic equilibrium. Concretely, one can have

(5) $$\begin{array}{c} S^{*}=\frac{\left[m\beta \left(m+\sigma +\mu \right)+\mu \left(m+\mu \right)\left(m+\sigma \right)\left(R_{0}-1\right)\right]N^{*}}{\beta \left[\left(m+\mu \right)\left(m+\sigma \right)\left(R_{0}-1\right)+m\left(m+\sigma +\mu \right)\right]}, \end{array}$$

(6) $$\begin{array}{c} E^{*}=\frac{\left[r\left(1-N^{*}/k\right)-m\right]\left(m+\mu \right)N^{*}}{\sigma \mu }, \end{array}$$

(7) $$\begin{array}{c} I^{*}=\frac{\left(m+\mu \right)\left(m+\sigma \right)\left(R_{0}-1\right)N^{*}}{\beta \left(m+\sigma +\mu \right)}, \end{array}$$

(8) N∗=(k[βm(m+σ+μ)(Rd−1)  +μ(m+σ)(m+μ)(1−R0)])×(rβ(m+σ+μ))−1,

where *R*_*d*_ = *r*/*m* is the basic demographic reproduction number and *R*_0_ = *βσ*/(*m* + *σ*)(*m* + *μ*) is the basic reproduction number which can be obtained by the next-generation matrix method [33–35]. The introduction of the basic demographic reproduction number can be found in [36].

Moreover, from (6) and (7), the conditions of the endemic equilibrium to exist are *R*_0_ > 1 and *R*_*d*_ > *R*_1_, where *R*_1_ = 1 + *μ*(*m* + *μ*)(*m* + *σ*)(*R*_0_ − 1)/*mβ*(*m* + *σ* + *μ*). So, we can obtain the following theorems.

**Theorem 2**

The system (1) has three equilibria: origin *O*, disease-free equilibrium *E*_0_, and the endemic equilibrium *E*_∗_. *O* always exists; if *R*_*d*_ > 1, *E*_0_ exists; if *R*_0_ > 1 and *R*_*d*_ > *R*_1_, *E*_∗_ exists.

**Theorem 3**

When *R*_*d*_ > 1 and *R*_0_ < 1, *E*_0_ is globally asymptotically stable.

**Proof**

By [33–35], we know that *E*_0_ is locally asymptotically stable. Now we define a Lyapunov function

(9) $$\begin{array}{c} V=E+\frac{m+\sigma }{\sigma }I\geq 0. \end{array}$$

When *R*_*d*_ > 1 and *R*_0_ < 1, the Lyapunov function satisfies

(10) V˙=E˙+m+σσI˙=βSIN−(m+σ)(m+μ)σI≤[β−(m+σ)(m+μ)σ]I=(m+σ)(m+μ)σ[R0−1]I≤0.

Moreover, $\dot{V}=0$ only hold when *I* = 0. It is easy to verify that the disease-free equilibrium point *E*_0_ is the only fixed point of the system. Hence, applying the Lyapunov-LaSalle asymptotic stability theorem in [37, 38], the disease-free equilibrium point *E*_0_ is globally asymptotically stable.

Since the proof of the stability of equilibria *O* and *E*_∗_ is more difficult, we only give some numerical results.

**Figure 1:**
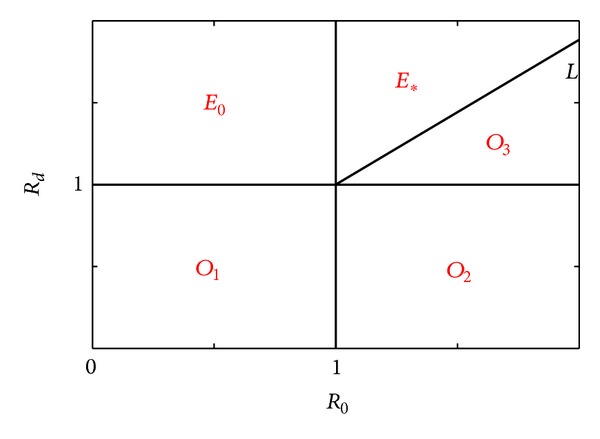
*R*_*d*_ in terms of *R*_0_. The region of *O*_1_, *O*_2_, and *O*_3_ is the basin of attraction of equilibrium *O*; the region of *E*_0_ is the basin of attraction of equilibrium *E*_0_; the region of *E*_∗_ is the basin of attraction of equilibrium *E*_∗_; the line *L* is *R*_*d*_ = *R*_1_ = 1 + *μ*(*m* + *μ*)(*m* + *σ*)(*R*_0_ − 1)/*mβ*(*m* + *σ* + *μ*).

**Figure 2:**
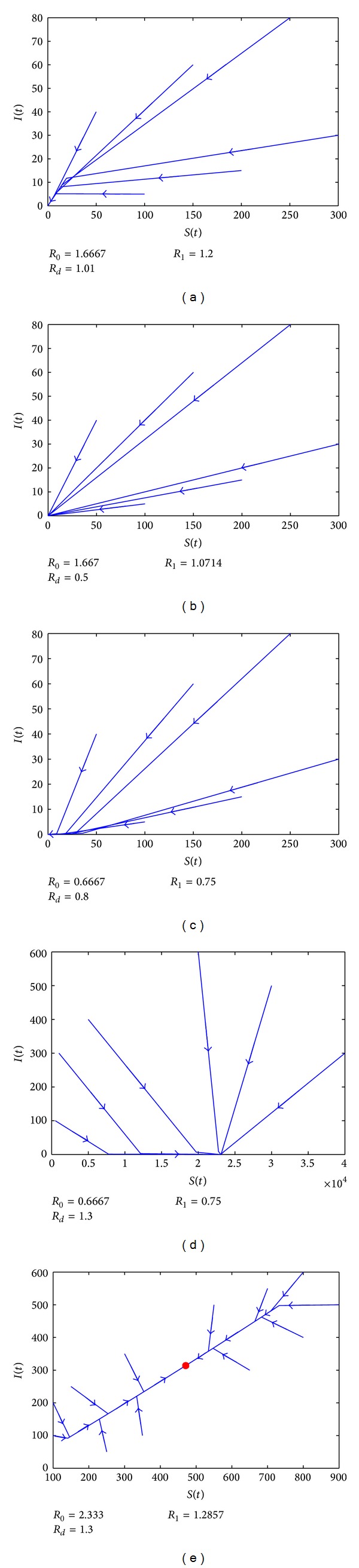
The phase curves of the system under different initial conditions. (a) In the region *O*_3_ with *r* = 0.101 and *β* = 0.5; (b) in the region *O*_2_ with *r* = 0.05 and *β* = 0.35; (c) in the region *O*_1_ with *r* = 0.08 and *β* = 0.2; (d) in the region *E*_0_ with *r* = 0.13 and *β* = 0.2; (e) in the region *E*_∗_ with *r* = 0.13 and *β* = 0.7. The value of other parameters can be seen in Table 1.

In sum, we can show the respective basins of attraction of the three equilibria which can be seen in Figure 1 and confirmed in Figure 2.

1. When *R*_*d*_ < 1, *O* is stable; see Figures 2(b) and 2(c).
2. When *R*_*d*_ > 1 and *R*_0_ < 1, *E*_0_ is stable; see Figure 2(d).
3. When *R*_*d*_ > 1, *R*_0_ > 1, and *R*_*d*_ < *R*_1_, *O* is stable; see Figure 2(a).
4. When *R*_*d*_ > 1, *R*_0_ > 1, and *R*_*d*_ > *R*_1_, *E*_∗_ is stable; see Figure 2(e).

## 3. Nonautonomous Model and Analysis

### 3.1. The Basic Reproduction Number

Now, we consider the non-autonomous case of the model (1) when the transmission rate is periodic, which is given as follows:

(11) $$\begin{array}{c} \frac{dS}{dt}=rN\left(1-\frac{N}{k}\right)-\frac{\beta \left(t\right)SI}{N}-mS,\\ \frac{dE}{dt}=\frac{\beta \left(t\right)SI}{N}-\sigma E-mE,\\ \frac{dI}{dt}=\sigma E-mI-\mu I, \end{array}$$

where *β*(*t*) is a periodic function which is proposed by [39]. In the subsequent section, we will discuss the dynamical behavior of the system (11).

For system (11), firstly we can give the basic reproduction number *R*_0_. According to the basic reproduction number under non-autonomous system, we can refer to the method of [40, 41]. From the last section, we know that system (11) has one disease-free equilibrium *E*_0_ = (*N* ^0^, 0,0), where *N* ^0^ = (1 − *m*/*r*)*k*. By giving a new vector *x* = (*E*, *I*), we have

(12) $$\begin{array}{c} F=\left(\begin{array}{c} \frac{\beta \left(t\right)SI}{N}\\ 0 \end{array}\right),\;\;V=\left(\begin{array}{c} mE+\sigma E\\ mI+\mu I-\sigma E \end{array}\right),\\ V^{-}=\left(\begin{array}{c} mE+\sigma E\\ mI+\mu I \end{array}\right),\;\;V^{+}=\left(\begin{array}{c} 0\\ \sigma E \end{array}\right). \end{array}$$

Taking the partial derivative of the above vectors about variables *E*, *I* and substituting the disease-free equilibrium, we have

(13) $$\begin{array}{c} F\left(t\right)=\left(\begin{array}{cc} 0 & \beta \left(t\right)\\ 0 & 0 \end{array}\right),\\ V\left(t\right)=\left(\begin{array}{cc} m+\sigma & 0\\ -\sigma & m+\mu \end{array}\right). \end{array}$$

According to [41], denote *C*_*ω*_ to be the ordered Banach space of all *ω*-periodic functions from ℝ to ℝ^4^ which is equipped with the maximum norm ||·|| and the positive cone *C*_*ω*_^+^ : = {*ϕ* ∈ *C*_*ω*_ : *ϕ*(*t*) ≥ 0, ∀*t* ∈ ℝ_+_}. Over the Banach space, we define a linear operator *L* : *C*_*ω*_ → *C*_*ω*_ by

(14) (Lϕ)(t)=∫0∞Y(t,t−a)F(t−a)ϕ(t−a)da, ∀t∈ℝ+, ϕ∈Cω,

where *L* is called the next infection operator and the interpretation of *Y*(*t*, *t* − *a*), *ϕ*(*t* − *a*) can be seen in [41]. Then the spectral radius of *L* is defined as the basic reproduction number

(15) $$\begin{array}{c} R_{0}:=\rho \left(L\right). \end{array}$$

In order to give the expression of the basic reproduction number, we need to introduce the linear *ω*-periodic system

(16) $$\begin{array}{c} \frac{dw}{dt}=\left[-V\left(t\right)+\frac{F\left(t\right)}{\lambda }\right]w,\;t\in {\mathbb{R}}_{+}, \end{array}$$

with parameter *λ* ∈ ℝ. Let *W*(*t*, *s*, *λ*), *t* ≥ *s*, be the evolution operator of system (16) on ℝ^2^. In fact, *W*(*t*, *s*, *λ*) = Φ_(*F*/*λ*)−*V*_(*t*), and Φ_*F*−*V*_(*t*) = *W*(*t*, 0,1), for all *t* ≥ 0. By Theorems 2.1 and 2.2 in [41], the basic reproduction number also can be defined as *λ*_0_ such that *ρ*(Φ_(*F*/*λ*_0_)−*V*_(*ω*)) = 1, which can be straightforward to calculate.

### 3.2. Global Stability of the Disease-Free Equilibrium

**Theorem 4**

The disease-free equilibrium *E*_0_ is globally asymptotically stable when *R*_0_ < 1 and *R*_*d*_ > 1.

**Proof**

Theorem 2.2 in [41] implies that *E*_0_ is locally asymptotically stable when *R*_0_ < 1 and *R*_*d*_ > 1. So we only need to prove its global attractability. It is easy to know that *S*(*t*) ≤ *N* ^0^ = (1 − (*m*/*r*))*k*. Thus,

(17) $$\begin{array}{c} \frac{dE}{dt}\leq \beta \left(t\right)I-\left(m+\sigma \right)E,\\ \frac{dI}{dt}=\sigma E-mI-\mu I. \end{array}$$

The right comparison system can be written as

(18) $$\begin{array}{c} \frac{dE}{dt}=\beta \left(t\right)I-\left(m+\sigma \right)E,\\ \frac{dI}{dt}=\sigma E-mI-\mu I; \end{array}$$

that is,

(19) $$\begin{array}{c} \frac{dh}{dt}=\left(F\left(t\right)-V\left(t\right)\right)h\left(t\right),\;\;\;h\left(t\right)=\left(E\left(t\right),I\left(t\right)\right). \end{array}$$

For (19), Lemma 2.1 in [42] shows that there is a positive *ω*-periodic function $\hat{h}{\left(t)=(E(t),I(t)\right)}^{T}$ such that $h(t)=e^{pt}\hat{h}(t)$ is a solution of system (18), where *p* = (1/*ω*)ln⁡*ρ*(Φ_*F*−*V*_(*ω*)). By Theorem 2.2 in [41], we know that when *R*_0_ < 1 and *R*_*d*_ > 1, *ρ*(Φ_*F*−*V*_(*ω*)) < 1 and *p* < 0, which implies *h*(*t*) → 0 as *t* → *∞*. Therefore, the zero solution of system (18) is globally asymptotically stable. By the comparison principle [43] and the theory of asymptotic autonomous systems [44], when *R*_0_ < 1 and *R*_*d*_ > 1, *E*_0_ is globally attractive. Therefore, the proposition that *E*_0_ is globally asymptotically stable holds.

### 3.3. Existence of Positive Periodic Solutions

Before the proof of the existence of positive periodic solutions, we firstly introduce some denotations. Let *u*(*t*, *x*_0_) be the solution of system (11) with the initial value *x*_0_ = (*S*(0), *E*(0), *I*(0)). By the fundamental existence-uniqueness theorem [45], *u*(*t*, *x*_0_) is the unique solution of system (11) with *u*(0, *x*_0_) = *x*_0_.

Next, we need to introduce the Poincaré map *P* : *X* → *X* associated with system (11); that is,

(20) $$\begin{array}{c} P\left(x_{0}\right)=u\left(\omega ,x_{0}\right),\;\forall x_{0}\in X, \end{array}$$

where *ω* is the period.  Theorem 1 implies that *X* is positively invariant and *P* is a dissipative point.

Now, we introduce two subsets of *X*, *X*_0_ : = {(*S*, *E*, *I*) ∈ *X* : *E* > 0, *I* > 0} and ∂*X*_0_ = *X*∖*X*_0_.

**Lemma 5**

(a) When *R*_0_ > 1 and *r* > *m* + *μ*, there exists a *δ* > 0 such that when

(21) $$\begin{array}{c} ||\left(S\left(0\right),E\left(0\right),I\left(0\right)\right)-E_{0}||\leq \delta \end{array}$$

for any (*S*(0), *E*(0), *I*(0)) ∈ *X*_0_, one has

(22) $$\begin{array}{c} \underset{m\to \infty }{\mathrm{limsup}}d\left[P^{m}\left(S\left(0\right),E\left(0\right),I\left(0\right)\right),E_{0}\right]\geq \delta , \end{array}$$

where *E*_0_ = (*N* ^0^, 0,0).

(b) When *R*_0_ > 1 and *r* > *m* + *μ*, there exists a *δ* > 0 such that when

(23) $$\begin{array}{c} ||\left(S\left(0\right),E\left(0\right),I\left(0\right)\right)-O||\leq \delta \end{array}$$

for any (*S*(0), *E*(0), *I*(0)) ∈ *X*_0_, one has

(24) $$\begin{array}{c} \underset{m\to \infty }{\mathrm{limsup}}d\left[P^{m}\left(S\left(0\right),E\left(0\right),I\left(0\right)\right),O\right]\geq \delta , \end{array}$$

where *O* = (0,0, 0).

**Proof**

(a) By Theorem 2.2 in [41], we know that when *R*_0_ > 1, *ρ*(Φ_*F*−*V*_(*ω*)) > 1. So there is a small enough positive number *ϵ* such that *ρ*(Φ_*F*−*V*−*M*_*ϵ*__(*ω*)) > 1, where

(25) $$\begin{array}{c} M_{\epsilon }=\left(\begin{array}{cc} 0 & \frac{\beta \left(t\right)\epsilon I}{N^{0}}\\ 0 & 0 \end{array}\right). \end{array}$$

If proposition (a) does not hold, there is some (*S*(0), *E*(0), *I*(0)) ∈ *X*_0_ such that

(26) $$\begin{array}{c} \underset{m\to \infty }{\mathrm{limsup}}d\left(P^{m}\left(S\left(0\right),E\left(0\right),I\left(0\right)\right),E_{0}\right)<\delta . \end{array}$$

We can assume that for all *m* ≥ 0, *d*(*P* ^*m*^(*S*(0), *E*(0), *I*(0)), *E*_0_) < *δ*. Applying the continuity of the solutions with respect to the initial values,

(27) ||u(t,Pm(S(0),E(0),I(0)))−u(t,E0)||≤ϵ,∀m≥0, ∀t1∈[0,ω].

Let *t* = *mω* + *t*_1_, where *t*_1_ ∈ [0, *ω*] and *m* = [*t*/*ω*]. *m* = [*t*/*ω*] is the greatest integer which is not more than *t*/*ω*. Then, for any *t* ≥ 0,

(28) ||u(t,(S(0),E(0),I(0)))−u(t,E0)|| =||u(t1,Pm(S(0),E(0),I(0)))−u(t1,E0)||≤ϵ.

So *N* ^0^ − *ϵ* ≤ *S*(*t*) ≤ *N* ^0^ + *ϵ*. Then, when limsup⁡_*m*→*∞*_ *d*(*P* ^*m*^(*S*(0), *E*(0), *I*(0)), *E*_0_) < *δ*,

(29) $$\begin{array}{c} \frac{dE}{dt}\geq \beta \left(t\right)I-\frac{\beta \left(t\right)\epsilon I}{N^{0}}-\left(m+\sigma \right)E,\\ \frac{dI}{dt}=\sigma E-mI-\mu I. \end{array}$$

Thus, we can study the right linear system

(30) $$\begin{array}{c} \frac{dE}{dt}=\beta \left(t\right)I-\frac{\beta \left(t\right)\epsilon I}{N^{0}}-\left(m+\sigma \right)E,\\ \frac{dI}{dt}=\sigma E-mI-\mu I. \end{array}$$

For the system (30), there exists a positive *ω*-periodic function $\hat{g}{\left(t)=(E(t),I(t)\right)}^{T}$ such that $g(t)=e^{pt}\hat{g}(t)$ is a solution of system (11), where *p* = (1/*ω*)ln⁡*ρ*(Φ_*F*−*V*−*Mϵ*_(*ω*)). When *R*_0_ > 1, *ρ*(Φ_*F*−*V*−*Mϵ*_(*ω*)) > 1, which means that when *g*(0) > 0, *g*(*t*) → *∞* as *t* → *∞*. By the comparison principle [43], when *E*(0) > 0, *I*(0) > 0, *E*(*t*) → *∞*, *I*(*t*) → *∞* as *t* → *∞*. There appears a contradiction. Thus, the proposition (a) holds.

(b) When *R*_0_ > 1 and *r* > *m* + *μ*, we have

(31) dNdt=rN(1−Nk)−mN−μI≥[r(1−Nk)−m−μ]N>0.

So if *R*_*d*_ > 1, *N* → *N* ^0^ for *N*(0) > 0 as *t* → +*∞*; that is, *W* ^*S*^(*O*)∩*X*_0_ = *∅*.

**Theorem 6**

When *R*_0_ > 1 and *r* > *m* + *μ*, there exists a *δ* > 0 such that any solution (*S*(*t*), *E*(*t*), *I*(*t*)) of system (11) with initial value (*S*(0), *E*(0), *I*(0))∈{(*S*, *E*, *I*) ∈ *X* : *E* > 0, *I* > 0} satisfies

(32) $$\begin{array}{c} \underset{t\to \infty }{\mathrm{liminf}}\;E\left(t\right)\geq \delta ,\;\;\underset{t\to \infty }{\mathrm{liminf}}\;I\left(t\right)\geq \delta , \end{array}$$

and system (11) has at least one positive periodic solution.

**Proof**

From system (11),

(33) S(t)=e−∫0t(β(s)I(s)/N+m)ds ×[S(0)+∫0trN(s)(1−N(s)k)       ×e∫0s(β(c)I(c)/N(c)+m)dcds]>0, ∀t>0,

(34) E(t)=e−(m+σ)t[E(0)+∫0tβ(s)S(s)I(s)N(s)e(m+σ)sds]>0, ∀t>0,

(35) I(t)=e−(m+μ)t[I(0)+∫0tσE(s)e(m+μ)sds]>0, ∀t>0,

for any (*S*(0), *E*(0), *I*(0)) ∈ *X*_0_, which shows that *X*_0_ is positively invariant. Moreover, it is obvious to see that ∂*X*_0_ is relatively closed in *X*. Denote

(36) M∂={(S(0),E(0),I(0))∈∂X0:  Pm(S(0),E(0),I(0))∈∂X0,∀m≥0}.

Next, we prove that

(37) $$\begin{array}{c} M_{\partial }=\left\{\left(S,0,0\right)\in X:S\geq 0\right\}. \end{array}$$

We only need to show that *M*_∂_⊆{(*S*, 0,0) ∈ *X* : *S* ≥ 0}, which means that for any (*S*(0), *E*(0), *I*(0))∈∂*X*_0_, *E*(*mω*) = *I*(*mω*) = 0, for all *m* ≥ 0. If it does not hold, there exists a *m*_1_ ≥ 0 such that (*E*(*m*_1_ *ω*), *I*(*m*_1_ *ω*))^*T*^ > 0. Taking *m*_1_ *ω* as the initial time and repeating the processes as in (33)–(35), we can have that (*S*(*t*), *E*(*t*), *I*(*t*))^*T*^ > 0, for all *t* > *m*_1_ *ω*. Thus, (*S*(*t*), *E*(*t*), *I*(*t*)) ∈ *X*_0_, for all *t* > *m*_1_ *ω*. There appears a contradiction, which means that the equality (37) holds. Therefore, *E*_0_ is acyclic in ∂*X*_0_. Obviously, when *R*_0_ > 1 and *r* > *m* + *μ*, *O* is acyclic in ∂*X*_0_.

Furthermore, by Lemma 5, *E*_0_ = (*N* ^0^, 0,0) and *O* = (0,0, 0) are isolated invariant sets in *X*, *W* ^*S*^(*E*_0_)∩*X*_0_ = *∅*, and *W* ^*S*^(*O*)∩*X*_0_ = *∅*. By Theorem 1.3.1 and Remark 1.3.1 in [46], it can be obtained that *P* is uniformly persistent with respect to (*X*_0_, ∂*X*_0_); that is, there exists a *δ* > 0 such that any solution (*S*(*t*), *E*(*t*), *I*(*t*)) of system (11) with the initial value (*S*(0), *E*(0), *I*(0))∈{(*S*, *E*, *I*) ∈ *X* : *E* > 0, *I* > 0} satisfies

(38) $$\begin{array}{c} \underset{t\to \infty }{\mathrm{liminf}}\;E\left(t\right)\geq \delta ,\;\;\underset{t\to \infty }{\mathrm{liminf}}\;I\left(t\right)\geq \delta . \end{array}$$

Applying Theorem 1.3.6 in [46], *P* has a fixed point

(39) $$\begin{array}{c} \left(S^{*}\left(0\right),E^{*}\left(0\right),I^{*}\left(0\right)\right)\in X_{0}. \end{array}$$

From (33), we know S* > 0, for all *t* ∈ [0, *ω*]. S*(*t*) is also more than zero for all *t* > 0 due to the periodicity. Similarly, for all *t* ≥ 0, E*(*t*) > 0, I*(*t*) > 0. Therefore, it can be obtained that one of the positive *ω*-periodic solutions of system (11) is (S*(*t*), E*(*t*), I*(*t*)).

### 3.4. Numerical Simulations

Firstly, we give some notations. If *g*(*t*) is a periodic function with period *ω*, we define $\overset{-}{g}=(1/\omega )\int_{0}^{\omega }g(t)dt$, *g* ^*l*^ = min⁡_*t*∈[0,*ω*]_ *g*(*t*), *g* ^*u*^ = max⁡_*t*∈[0,*ω*]_ *g*(*t*). As described in the previous section,

(40) $$\begin{array}{c} R_{1}\left(t\right)=1+\frac{\mu \left(m+\mu \right)\left(m+\sigma \right)\left(R_{0}-1\right)}{m\beta \left(t\right)\left(m+\sigma +\mu \right)}. \end{array}$$

So *R*_1_^*l*^ = 1 + *μ*(*m* + *μ*)(*m* + *σ*)(*R*_0_^*l*^ − 1)/*mβ* ^*u*^(*m* + *σ* + *μ*), *R*_1_^*u*^ = 1 + *μ*(*m* + *μ*)(*m* + *σ*)(*R*_0_^*u*^ − 1)/*mβ* ^*l*^(*m* + *σ* + *μ*).

**Figure 3:**
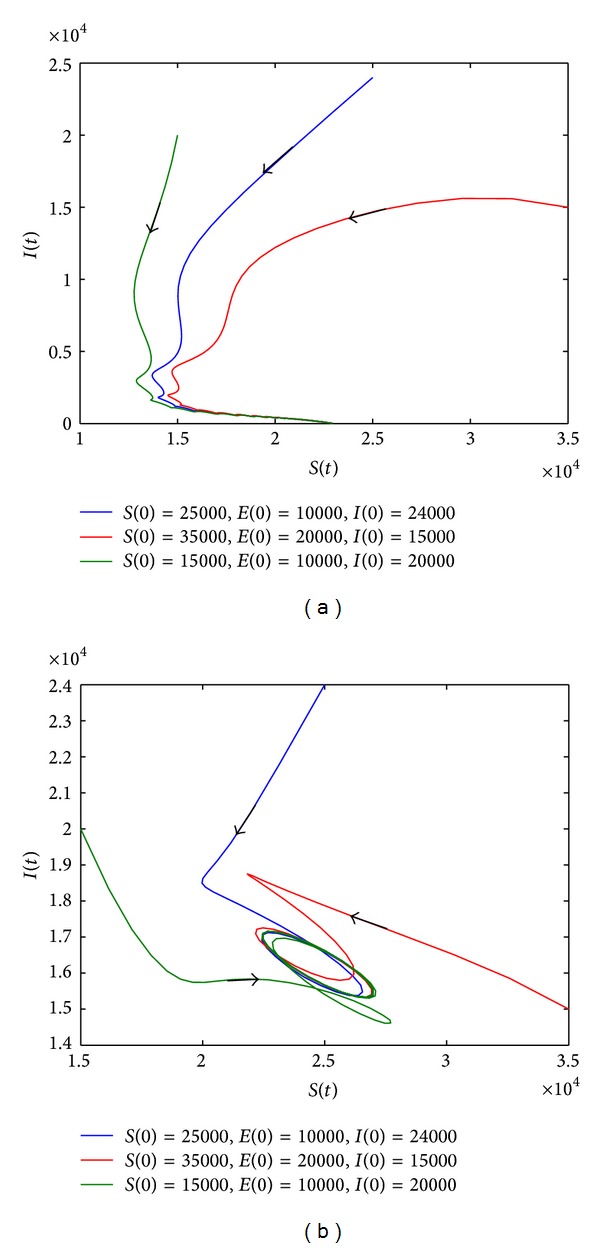
Phase plane of *S*(*t*) and *I*(*t*). (a) When the parameter values are *r* = 0.13, *a* = 0.3, and *b* = 0.2, *R*_0_ = 0.9029 < 1, *R*_*d*_ = 1.3 > 1. (b) When the parameter values are *r* = 0.3, *a* = 0.7, and *b* = 0.2, *R*_0_ = 2.1067 and *r* > *m* + *μ*. The value of other parameters can be seen in Table 1.

In this section, we adopt *β*(*t*) = *a*[1 + *b*sin(*πt*/10)]. Then, applying the numerical simulation to verify the above solution, we give the following conclusion:

1. when *R*_*d*_ < 1, *O* is stable;
2. when *R*_*d*_ > 1 and *R*_0_ < 1, *E*_0_ is stable; see Figure 3(a);
3. when *R*_0_ > 1 and *r* > *m* + *μ*, system (11) has at least one positive periodic solution; see Figure 3(b).

**Figure 4:**
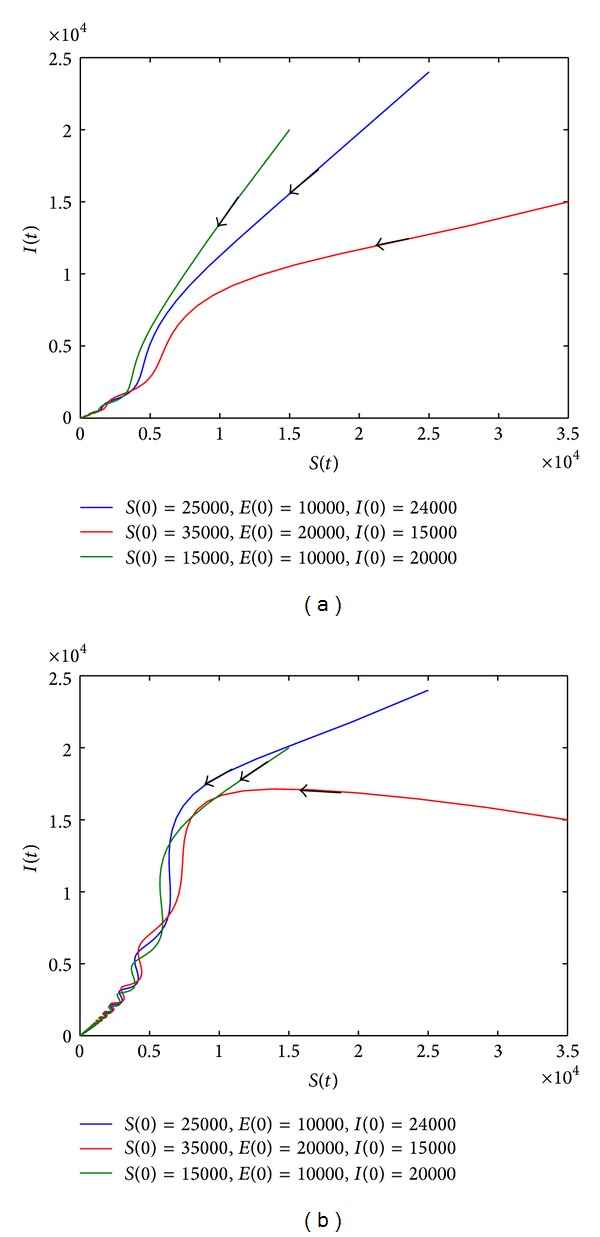
Phase plane of *S*(*t*) and *I*(*t*). (a) When the parameter values are *r* = 0.11, *a* = 0.9, *b* = 0.2 and *μ* = 0.3, *R*_0_ = 1.4991, *R*_*d*_ = 1.1 > 1, *R*_*d*_ < *R*_1_^*u*^ = 1.4159, and *R*_*d*_ < *R*_1_^*l*^ = 1.2773. (b) When the parameter values are *r* = 0.126, *a* = 0.7, *b* = 0.2, and *μ* = 0.3, *R*_0_ = 2.1067, *R*_*d*_ = 1.26 > 1, *R*_*d*_ < *R*_1_^*u*^ = 1.2964, and *R*_*d*_ > *R*_1_^*l*^ = 1.1976. The value of other parameters can be seen in Table 1.

**Figure 5:**
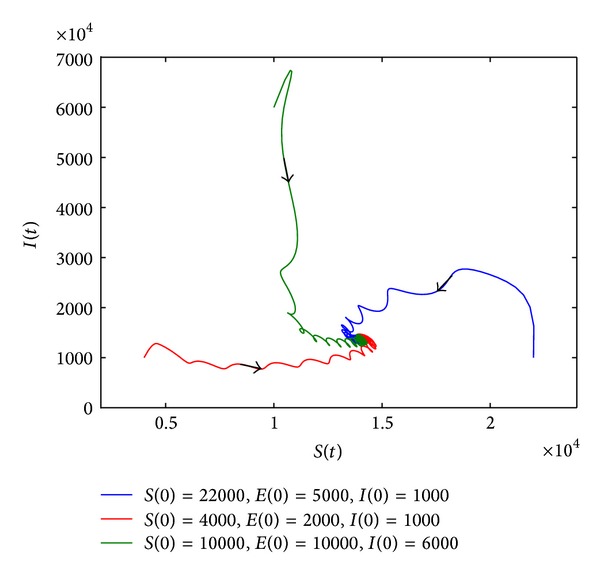
Phase plane of *S*(*t*) and *I*(*t*). When the parameter values are *r* = 0.13, *a* = 0.36, and *b* = 0.2, *R*_0_ = 1.0834, *R*_*d*_ = 1.3 > 1, *R*_*d*_ > *R*_1_^*u*^ = 1.0435, and *R*_*d*_ > *R*_1_^*l*^ = 1.029. The value of other parameters can be seen in Table 1.

We can give more results about the conditions of existence of the positive periodic solution.

(1′) When *R*_*d*_ > 1, *R*_0_ > 1, and *R*_*d*_ < *R*_1_^*u*^, *O* is stable; see Figures 4(a) and 4(b).
(2′) When *R*_*d*_ > 1, *R*_0_ > 1, and *R*_*d*_ > *R*_1_^*u*^, system (11) has at least one positive periodic solution, see Figure 5.

By numerical simulations, we can give that the conditions which ensure the existence of positive periodic solution are *R*_0_ > 1 and *r* > *m* + *μ* or *R*_*d*_ > 1, *R*_0_ > 1, and *R*_*d*_ > *R*_1_^*u*^. In fact, *R*_*d*_ > 1, *R*_0_ > 1, and *R*_*d*_ > *R*_1_^*u*^ are the sufficient conditions for *R*_0_ > 1 and *r* > *m* + *μ*. As a result, the conditions *R*_0_ > 1 and *r* > *m* + *μ* are broader.

## 4. Discussion

This paper considers a logistic growth system whose birth process incorporates density-dependent effects. This type of model has a rich dynamical behavior and practical significance. By analyzing its equilibria and respective attractive region, we find that the dynamical behavior of a disease will be determined by two thresholds *R*_0_ and *R*_*d*_. Only *R*_0_ > 1 cannot promise the existence of the endemic equilibrium which also needs *R*_*d*_ > *R*_1_. When *R*_0_ > 1 and *R*_*d*_ < *R*_1_, the solutions of the system (1) will tend to the origin *O*. It is caused by the phenomenon that the death number due to disease cannot be supplemented by the birth number promptly. Finally, all people are infected and die out. The fact interpreted by this model is more reasonable. Theoretically, we prove the global asymptotic stability of the disease-free equilibrium and give respective attractive regions of equilibria. 

Seasonally effective contact rate is the most common form which may be related to various factors, and thus this paper studies the corresponding non-autonomous system which is obtained by changing the constant transmission rate of the above system into the periodic transmission rate. For the periodic systems, their dynamical behaviors, especially the basic reproduction number, have been investigated in depth by [41, 47–55] which provide many methods that we can utilize. For the obtained periodic model, by analyzing the global asymptotic stability of the disease-free equilibrium and the existence of positive periodic solution, we have the similar results as the autonomous system. The dynamic behavior of disease will be decided by two conditions *R*_0_ > 1 and *r* > *m* + *μ* that show that when the disease is prevalent, the birth rate should be larger than the death rate to guarantee the sustainable growth of population. Otherwise, the population will disappear. In addition, we will evaluate and compare the basic reproduction number *R*_0_ and the average basic reproduction number ${\overset{-}{R}}_{0}$ which has been adopted by [27, 56–59]. In this paper, we can calculate the average reproduction number

(41) $$\begin{array}{c} {\overset{-}{R}}_{0}=\frac{\overset{-}{\beta }\sigma }{\left(m+\sigma \right)\left(m+\mu \right)}, \end{array}$$

where $\overset{-}{\beta }=\left(1/20\right)\int_{0}^{20}\beta (t)dt$. When *r* = 0.13, *a* = 0.3, and *b* = 0.2, we know that *R*_0_ = 0.9029 and ${\overset{-}{R}}_{0}=1$. When *r* = 0.13, *k* = 100000, *a* = 0.36, *b* = 0.2, and *m* = 0.1, *σ* = 0.2, *μ* = 0.1, then *R*_0_ = 1.0834 and ${\overset{-}{R}}_{0}=1.2$. In that sense, it is confirmed that the basic reproduction number *R*_0_ defined by [40] is more accurate than the average reproduction number ${\overset{-}{R}}_{0}$ which overestimates the risk of disease.

It should be noted that we live in a spatial world and it is a natural phenomenon that a substance goes from high density regions to low density regions. As a result, epidemic models should include spatial effects. In a further study, we need to investigate spatial epidemic models with seasonal factors.

## Figures and Tables

**Table 1:** Descriptions and values of parameters in model (1).

Parameter	Interpretation	Value
*r*	The intrinsic growth rate	
*k*	The carrying capacity	100000
*β*	The transmission rate	
*m*	The natural mortality rate	0.1
*σ*	Clinical outcome rate	0.2
*μ*	The disease-induced mortality rate	0.1
*a*	The baseline contact rate	
*b*	The magnitude of forcing	
